# The Effect of Resistance Training and Ursolic Acid on the PI3K‐AKT‐mTOR Pathway in Aged Diabetic Rats: A Comparative Study

**DOI:** 10.1002/agm2.70071

**Published:** 2026-05-25

**Authors:** Ashkan Sadeghi, Safoura Alizade, Mohammad Faramarzi, Mostafa Rahimi, Rohit Kumar Thapa

**Affiliations:** ^1^ Department of Sport Sciences Shahrekord University Shahrekord Iran; ^2^ Department of Exercise Physiology, Faculty of Sport Sciences University of Isfahan Isfahan Iran; ^3^ Symbiosis School of Sports Sciences Symbiosis International (Deemed University) Pune India

**Keywords:** glucose metabolism disorders, insulin resistance, muscle atrophy, sarcopenia, type 2 diabetes mellitus

## Abstract

**Objectives:**

Sarcopenia, characterized by age‐related muscle loss, worsens in diabetes due to anabolic resistance. Ursolic acid (UA), a natural compound with anabolic and anti‐catabolic effects, may mitigate sarcopenia by enhancing anabolic pathways. This study examined the effects of 8 weeks of resistance training and UA supplementation on PI3K‐AKT‐mTORC1 pathway proteins in muscle tissue of aged diabetic rats.

**Methods:**

Fifty 21‐month‐old Wistar rats were divided into five groups: healthy control, diabetic control, diabetic + resistance training, diabetic + UA, and diabetic + resistance training + UA. Type 2 diabetes was induced using a high‐fat diet and low‐dose STZ. Resistance training consisted of 8 weeks of ladder climbing at 60% MVCC, 5 days per week. UA was administered daily to the UA and combination groups. Protein expression was analyzed using Western blot.

**Results:**

AKT and mTORC1 or phosphorylated AKT levels did not differ significantly across groups. However, dephosphorylated PI3K (*p* = 0.011) and phosphorylated mTORC1 (*p* = 0.026) showed significant changes. PI3K expression decreased in diabetic, resistance training, and UA groups compared to controls, but not in the combination group. Phosphorylated mTORC1 was reduced in diabetic controls but maintained in the training, UA, and combination groups.

**Conclusion:**

Diabetes reduces PI3K and mTORC1 protein expression. Resistance training or UA alone improved mTORC1 expression, while their combination enhanced both PI3K and mTORC1, suggesting synergistic anabolic benefits. Combining UA with resistance training may counteract diabetes‐induced muscle loss.

## Graphical abstract

Resistance training combined with ursolic acid supplementation restores anabolic signaling (PI3K/mTORC1) suppressed by diabetes, helping preserve muscle mass. This strategy may offer a potential therapeutic approach to combat muscle atrophy and support healthy aging in diabetic conditions.

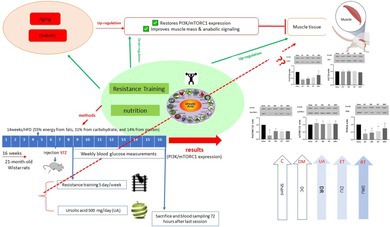

## 1 Introduction

Aging is a natural, gradual process resulting from cellular and molecular damage, leading to deterioration and functional decline in tissues [1, 2]. A sedentary lifestyle during aging is commonly associated with sarcopenia, an age‐related disorder characterized by significant reductions in muscle mass and strength [3]. Key indicators of sarcopenia include muscle atrophy and decreased muscle fiber number [4, 5]. Muscle atrophy is regulated by specific signaling pathways, with anabolic resistance being a major contributor [6, 7]. Notably, anabolic responses to dietary intake and exercise are significantly reduced in elderly individuals compared to younger adults [8]. Moreover, metabolic diseases such as diabetes exacerbate the risk of sarcopenia, muscle atrophy, and anabolic resistance [9, 10].

A major issue in aging‐related anabolic resistance is impaired muscle protein synthesis (MPS). The primary pathway regulating MPS is the PI3K‐AKT‐mTORC1 pathway, which is activated by resistance exercise and dietary intake [11, 12], leading to increased MPS and a positive net protein balance, reducing muscle atrophy risk [13]. The mTOR pathway, a key regulator of protein synthesis, also inhibits autophagy [14, 15] and facilitates cell division and gene transcription [16]. It comprises two complexes, mTORC1 and mTORC2, with mTORC1 playing the central role in response to resistance exercise [17]. Amino acids can activate mTOR through small G‐proteins [18], while growth factors such as insulin stimulate mTOR via a cascade involving IRS, PI3K, and AKT/PKB [19]. The PI3K‐AKT pathway governs skeletal muscle growth [20, 21, 22], with AKT acting upstream, primarily through mTOR signaling, which exerts its effects mainly through mTOR signaling, which is critical for hypertrophic adaptations induced by mechanical loading [23].

Resistance exercise activates the mTOR pathway, increases muscle protein synthesis (MPS), and mitigates muscle atrophy [24]. It also improves insulin sensitivity, which is particularly important for diabetic individuals [25]. Regular physical activity alleviates functional decline in elderly people with sarcopenia and enhances their ability to perform daily tasks [26]. Resistance training exerts mechanical stress on muscle tissue, activating satellite cells [27], stimulating collagen turnover [28], and increasing myofibrillar protein synthesis, thereby enhancing muscle mass and functionality [7, 29, 30]. Furthermore, it enlarges the cross‐sectional area of both type I and II muscle fibers, improving strength in the elderly [31, 32, 33].

Ursolic acid (UA), a pentacyclic triterpenoid found in various plants, especially apple peel [34, 35], has been shown to prevent muscle loss, improve insulin sensitivity, reduce inflammation, and stimulate muscle growth [36, 37]. UA also reduces expression of atrophy‐related proteins such as atrogin‐1 and MuRF1 [38, 39], and has anabolic effects on skeletal muscle [40, 41], suggesting its potential role in preventing sarcopenia [42]. Although the direct effects of UA supplementation on sarcopenia have not yet been fully investigated, existing evidence indicates that UA acts as a potent anabolic stimulus for MPS [40].

Considering the established anabolic effects of resistance exercise and UA, combining these interventions could represent an effective therapeutic strategy for mitigating muscle atrophy in elderly individuals with diabetes. However, to date, no study has explored the combined effects of UA supplementation and resistance exercise on PI3K‐AKT‐mTORC1 pathway proteins in muscle tissue. Therefore, the present study aimed to investigate this interaction in aged diabetic rats. We hypothesized that combined UA supplementation and resistance training would synergistically enhance PI3K‐AKT‐mTORC1 protein expression, thereby promoting muscle hypertrophy and improving metabolic outcomes.

## 2 Methods

### 2.1 Animals

For this laboratory study, 50 male Wistar rats aged 21 months (424 ± 35 g) were obtained from the Laboratory Animal Breeding Center of the Razi Institute. All experimental procedures complied with the ethical standards of the Animal Ethics Committee at Shahrekord University (Iran) and were conducted according to the Guide for the Care and Use of Laboratory Animals (Approval code: IR.SKU.REC.1399.001; ethics.research.ac.ir). Thus, all stages of the research were reviewed and approved by the institutional ethics committee. To allow adaptation to the laboratory environment and the experimenter, the rats were transferred to the Laboratory Animal Facility of Shahrekord University and housed under controlled conditions (temperature: 22°C ± 3°C; 12/12‐h light/dark cycle) with ad libitum access to food and water. After a one‐week acclimatization period, animals were randomly assigned to five groups (*n* = 10 per group) as follows: Healthy Control (Sham), Diabetic Control (DC), Diabetic + Resistance Exercise (DR), Diabetic + Ursolic Acid (DU), Diabetic + Resistance Exercise + Ursolic Acid (DRU).

### 2.2 Induction of Diabetes

Type 2 diabetes was induced using a high‐fat diet (HFD) combined with a low dose of streptozotocin (STZ), following the protocol of Zhang et al. [43]. The Sham group received a standard rodent diet containing 10% of energy from fat, 75% from carbohydrates, and 15% from protein (3.8 kcal/g). In contrast, the other groups were fed a HFD consisting of 55% of energy from fat, 31% from carbohydrates, and 14% from protein (5.2 kcal/g). Both groups maintained their respective diets for eight weeks. In the fourth week, rats in the HFD groups were injected intraperitoneally with STZ (30 mg/kg; Sigma‐Aldrich, St. Louis, MO, USA). STZ was freshly prepared in 0.1 M sodium citrate buffer (pH 4.4–4.5) and immediately injected to prevent degradation. One week after injection, blood samples were collected and glucose levels were measured [44]. Rats with blood glucose < 16.7 mmol/L received a second STZ injection (30 mg/kg). Sham rats received an equivalent volume of citrate buffer (0.25 mL/kg). Following the injections, all groups continued their respective diets. Four weeks later, rats with blood glucose concentrations > 16.7 mmol/L were considered diabetic and included in the study [45].

### 2.3 Determination of Maximum Voluntary Carrying Capacity (MVCC)

Rats in the resistance training groups were first familiarized with a vertical climbing ladder (110 cm height, 2 cm grid spacing, 85° incline) for one week (five sessions per week). Seventy‐two hours after the final familiarization session, each rat underwent MVCC testing. For the initial climb, a load equivalent to 75% of body weight was attached to the tail, and the rat was required to climb to the top of the ladder. After a 120‐s rest period, the load was progressively increased in 30 g increments until the rat could no longer complete the full climb. The test was terminated when the rat failed to voluntarily climb the entire ladder in three consecutive attempts. The heaviest successfully carried load over the full ladder length was recorded as the MVCC [46].

### 2.4 Resistance Training Protocol

The resistance training protocol involved ladder climbing with a weight attached to the tail. The ladder was 110 cm in length, set at an 80° incline, and equipped with 26 rungs spaced 2 cm apart. Training was conducted over eight weeks (five sessions per week), with at least 24 h of recovery between sessions. MVCC was assessed at baseline, week 4, and week 8 of the study. Training intensity was set at 60% of MVCC, with 14–20 climbs per session and one‐minute rest intervals between climbs (Table 1) [46].

**TABLE 1:** Resistance training protocol.

Number of sessions/week	Repetitions	Intensity (%)	Rest	Weeks
5	14–20	60% MVCC	1 min	8

Abbreviation: MVCC, maximal voluntary carrying capacity.

### 2.5 Ursolic Acid Supplementation Protocol

Ursolic acid (UA) was obtained from Healthy‐Aging Supplementation, a knowledge‐based company (Tehran, Iran; product code 9870). Aged rats received UA at a dose of 500 mg/kg (0.5%) incorporated into a high‐fat diet for 8 weeks [47, 48, 49]. The supplemented diet was prepared by Royan Company.

### 2.6 Blood Sampling and Tissue Extraction

Seventy‐two hours after the final training session, rats were anesthetized with an intraperitoneal injection of ketamine (90 mg/kg) and xylazine (10 mg/kg) [50]. The chest cavity was opened, and blood samples were collected directly from the heart. Samples were centrifuged at 1000× *g* for 10 min to separate the serum, which was immediately transferred to liquid nitrogen and stored at −80°C. Blood glucose was measured using an ELISA kit (Pars Azmoon, Tehran, Iran; cat. no. 5825; sensitivity: 0.1 mg/dL). Insulin levels were determined with an ELISA kit (Monobind, USA; cat. no. 300‐5825). Skeletal muscle samples (flexor hallucis longus, FHL) were excised, rinsed in physiological saline, placed in cryotubes, and snap‐frozen in liquid nitrogen. Serum and tissue samples were stored at −80°C until analysis.

Protein levels of PI3K, AKT, and mTOR were assessed by Western blotting using specific antibodies (Santa Cruz Biotechnology). Equal amounts of protein were separated by 12% SDS‐PAGE and transferred to PVDF membranes. Membranes were blocked for one hour, then incubated overnight at 4°C with primary antibodies. After washing with TBST (three times, 15 min each), membranes were incubated with HRP‐conjugated secondary antibodies for one hour. Protein bands were detected using enhanced chemiluminescence (ECL) and visualized on radiographic films.

Membranes were subsequently stripped and reprobed with anti‐β‐actin antibody (mouse monoclonal, SC‐4778, clone C4) as a loading control. Bands were re‐visualized, and densitometric quantification was performed using ImageJ Studio Lite software.

### 2.7 Statistical Analysis

Data were analyzed using SPSS software (version 25, IBM, New York, USA), and graphs were prepared with GraphPad Prism (version 8.4; GraphPad Software Inc., San Diego, CA, USA). Descriptive statistics, including mean ± standard deviation, were calculated. Between‐group differences were assessed using one‐way analysis of variance (ANOVA). When ANOVA revealed significant effects, Tukey's post hoc test was applied for pairwise comparisons. For MVCC, repeated‐measures ANOVA was conducted to evaluate within‐group and between‐group differences across time points. Statistical significance was set at *p* < 0.05.

## 3 Results

The changes in body weight, blood glucose, and insulin levels are summarized in Table 2. One‐way ANOVA revealed significant differences among groups for body weight (*p* = 0.001), glucose (*p* = 0.001), and insulin (*p* = 0.002). Tukey's post hoc test indicated that body weight was significantly lower in the DC (*p* = 0.001), DR (*p* = 0.004), DU (*p* = 0.038), and DRU (*p* = 0.005) groups compared to the sham group. Similarly, glucose levels were significantly higher in the DC (*p* = 0.001), DR (*p* = 0.001), DU (*p* = 0.001), and DRU (*p* = 0.001) groups relative to Sham. Insulin levels were significantly reduced in the DC group compared to Sham (*p* = 0.001), whereas DR (*p* = 0.001), DU (*p* = 0.001), and DRU (*p* = 0.001) groups exhibited significantly higher insulin levels compared to the DC.

**TABLE 2:** Comparison of changes in body weight, glucose, and insulin among study groups.

Variables	Sham group	DC group	DR group	DU group	DRU group	*F*	*p*
Weight change (g)	6.62 ± 5.15	−43.60 ± 26.60*	−42.80 ± 10.96*	−27.80 ± 7.24*	−41.37 ± 9.88*	10.919	0.001
Glucose	94.92 ± 9.11	357.25 ± 116.35*	360.60 ± 145.84*	322.31 ± 99.96*	434.41 ± 66.11*	16.008	0.001
Insulin	2.12 ± 0.81	0.22 ± 0.18*	0.53 ± 0.60#	0.54 ± 0.61#	0.57 ± 0.58#	11.334	0.001

Abbreviations: DC, diabetic control group; DR, diabetic + resistance exercise group; DRU, diabetic + resistance exercise + ursolic acid group; DU, diabetic + ursolic acid group; Sham, healthy control group.

* Significant difference compared to the healthy control group.

^#^ Significant difference compared to the diabetic control group.

**FIGURE 1:**
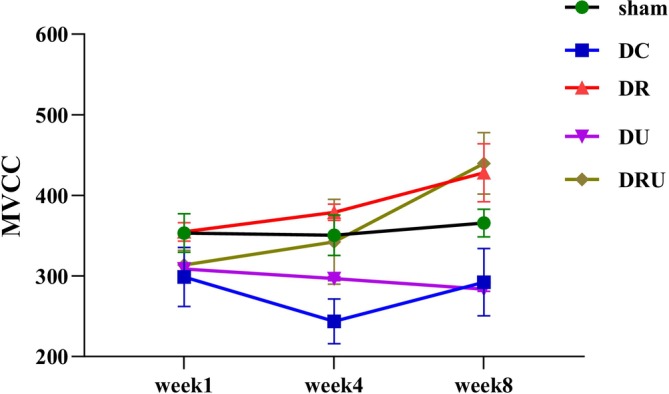
Maximum voluntary carrying capacity (MVCC) in aging diabetic rats at weeks 1, 4, and 8. Values are presented as mean ± SEM. Data were analyzed using repeated measures ANOVA, *n* = 10 per group.

A significant main effect of time was observed for MVCC (*p* = 0.001), along with a significant time × group interaction (*p* = 0.016). Post hoc analyses showed that MVCC was significantly greater in the DR group than in the DC group (*p* = 0.028). Additionally, within‐group comparisons revealed a significant increase in MVCC from week 1 to week 8 in the DRU group (*p* = 0.031) (Figure 1).

**FIGURE 2:**
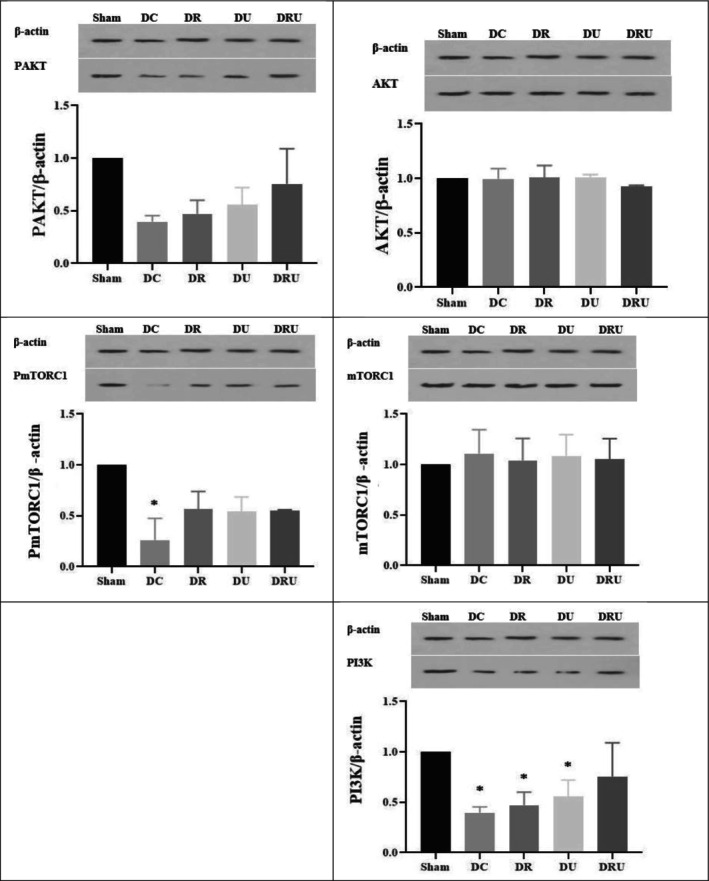
Western blot analysis of PI3K, AKT, mTORC1, and their phosphorylated forms (PAKT and PmTORC1) in the FHL muscle across study groups. DC, diabetic control; DR, diabetic + resistance Training; DRU, diabetic + resistance training + ursolic acid; DU, diabetic + ursolic acid; Sham, healthy control; *significant difference compared to the Sham group.

Changes in the expression of PI3K, AKT, and mTORC1 pathway proteins in the skeletal muscle of aged diabetic rats are shown in Figure 2. One‐way ANOVA revealed no significant differences among groups in the dephosphorylated forms of AKT (*p* = 0.683) and mTORC1 (*p* = 0.981), as well as the phosphorylated form of PAKT (*p* = 0.090). However, significant differences were observed for the dephosphorylated form of PI3K (*p* = 0.011) and phosphorylated mTORC1 (*p* = 0.026). Post hoc analysis indicated that PI3K expression in FHL muscle was significantly lower in the DC (*p* = 0.021), DR (*p* = 0.041), and DU (*p* = 0.044) groups compared to Sham. No significant difference was observed between the DRU and Sham groups (*p* = 0.011). Similarly, phosphorylated mTORC1 expression was significantly reduced in the DC group (*p* = 0.032) relative to Sham, whereas no significant differences were found for DR (*p* = 0.183), DU (*p* = 0.142), and DRU (*p* = 0.230) compared to the Sham group (Figure 2).

## 4 Discussion

The present study aimed to investigate the effect of resistance training, with or without ursolic acid (UA) supplementation, on the expression of PI3K, AKT, and mTORC1 proteins and their phosphorylated forms (PAKT and PmTORC1) in the skeletal muscle of aged diabetic rats. The results indicated that resistance training or UA supplementation alone did not significantly alter the expression of AKT and mTORC1 proteins, nor the phosphorylated form of PAKT. However, diabetes induction significantly reduced PmTORC1 levels and decreased PI3K expression. Notably, the combination of resistance training and UA supplementation prevented the reduction of PI3K expression compared to Sham. Hyperglycemia and chronic inflammation in diabetes impair IRS‐1 phosphorylation and PI3K recruitment, consequently reducing AKT activation and downstream mTORC1 signaling. This mechanism likely explains the suppression of PmTORC1 and PI3K observed in diabetic rats despite interventions [51, 52].

Previous studies have demonstrated that resistance training and UA supplementation, when applied separately, can activate hypertrophy‐related pathways and inhibit catabolic signaling [53, 54, 55]. For instance, Liang et al. reported that 8 weeks of resistance training activated the AKT/mTOR pathway in aged rats [56], while Fallah Mohammadi et al. observed increased mTOR expression following 8 weeks of combined resistance and endurance training in young rats [57]. These results partially contrast with the present findings. However, studies by Nemati et al. and Wang et al. support our observations, showing no significant changes in mTOR expression or reductions in P‐mTOR and P‐AKT in response to resistance training in young and aged rats, respectively [58, 59]. Furthermore, high‐intensity interval training for 4–8 weeks has been reported to increase PI3K/AKT/mTOR expression in type 2 diabetic rat models [60], and in humans, prolonged resistance training (16–24 weeks) can improve insulin sensitivity and muscle hypertrophy markers [61, 62]. High‐intensity resistance training [63], and high‐intensity interval training [60] have shown significant effects on hypertrophy pathways. These findings suggest that exercise intensity is a critical factor, and the moderate intensity used in the present study (60% MVCC) may have been insufficient to overcome diabetes‐induced anabolic resistance. Under normal conditions, resistance training activates the IGF‐1/PI3K/AKT/mTOR signaling cascade, stimulating protein synthesis via p70S6K and 4E‐BP1. In diabetes, however, anabolic resistance and impaired insulin signaling blunt this pathway's activation [64]. The moderate intensity applied in the present study may therefore have been insufficient to overcome this resistance.

Regarding UA supplementation, limited studies have examined its effects on PI3K/AKT/mTOR signaling. Previous evidence suggests that UA enhances muscle strength and mass while modulating hypertrophy‐ and atrophy‐related pathways. For instance, Ebert et al. reported increased muscle mass and strength following UA administration [65]. Similarly, Kunkel et al. demonstrated that 8 weeks of supplementation suppressed MuRF‐1 and atrogin‐1 expression, thereby attenuating muscle atrophy [38]. In line with this, Jeong et al. showed that 12 weeks of UA upregulated AKT/mTOR expression and simultaneously reduced expression of MURF‐1 and atrogin‐1 [66]. Conversely, Bakhtiari et al. observed a significant downregulation of AKT and IGF‐1 expression after 7 days of supplementation [67], while Ogasawara et al. reported enhanced mTOR gene expression [40]. Collectively, these findings indicate heterogeneous outcomes, many of which contrast with the present study. In addition to direct effects on muscle signaling, UA has been shown to improve insulin resistance in individuals with metabolic syndrome [39] and obese rats [48], thereby indirectly promoting PI3K/AKT activation and muscle protein synthesis. Mechanistically, UA is suggested to enhance IGF‐1 receptor signaling and AKT/mTOR activation while suppressing catabolic E3 ligases (MuRF‐1 and atrogin‐1). It also improves insulin sensitivity and reduces pro‐inflammatory cytokines, both of which facilitate PI3K/AKT pathway activation [68].

Despite these proposed mechanisms, the current study found no significant effects of UA supplementation on PI3K/AKT/mTOR proteins. Possible explanations include advanced diabetic pathology, insufficient intervention duration, or variations in supplementation protocol. Indeed, earlier studies have employed different dosages: 200 mg/kg [38], 150 mg/kg [39], and 0.5 g/kg [48] and reported positive outcomes, whereas the relatively higher dose (500 mg/kg) used in the present study yielded no significant results. Beyond dosage, factors such as timing of administration, method of delivery (with food, gavage, or in combination with olive oil) [38], and intervention length [66] may critically influence outcomes. Of note, research on the combined effects of resistance training and UA supplementation remains scarce. To date, only one study has examined this interaction, demonstrating that 8 weeks of concurrent resistance training and UA supplementation significantly elevated mTOR, IGF‐1, and irisin expression in healthy men [69]. In contrast, the present study did not observe significant effects of UA alone, but when combined with resistance training, PI3K expression was preserved, highlighting a potential synergistic benefit of the dual intervention. This suggests that UA may potentiate the anabolic effects of resistance training, particularly by counteracting diabetes‐induced impairments in muscle signaling.

The observed reduction in PI3K expression in the DC, DR, and DU groups indicates a suppression of the anabolic PI3K/AKT/mTOR pathway and diminished muscle protein synthesis. In contrast, the increased PI3K expression in the DRU group, achieved through the combination of ursolic acid supplementation and resistance training, highlights a synergistic activation of anabolic signaling and muscle protein synthesis. This synergy likely results from dual stimulation: mechanical loading induced by resistance training and the improved insulin sensitivity and anti‐inflammatory effects of ursolic acid, which together enhance mTORC1 phosphorylation and protein synthesis [41, 70]. The mTORC1 pathway, a central regulator of anabolic signaling and muscle protein synthesis, was significantly downregulated in the DC group compared to Sham, reflecting the adverse effects of diabetes on muscle homeostasis. Notably, interventions such as resistance training (DR), ursolic acid supplementation (DU), and their combination (DRU) restored or preserved mTORC1 expression at levels comparable to Sham. Restoration of mTORC1 suggests reactivation of its downstream targets (p70S6K and 4E‐BP1), which regulate translation initiation and muscle protein accretion [17, 40]. Taken together, these findings demonstrate the potential of resistance training and ursolic acid, individually or combined, to counteract the catabolic effects of diabetes and sustain anabolic signaling. Since diabetes is often associated with sarcopenia and impaired muscle protein synthesis, these interventions may represent promising therapeutic strategies to preserve muscle mass in diabetic populations.

## 5 Conclusion

This study demonstrated that diabetes significantly reduced PI3K expression and phosphorylated mTORC1 levels, indicating suppression of anabolic signaling and MPS. Resistance training or ursolic acid supplementation alone improved mTORC1 expression, whereas their combination effectively enhanced both PI3K and mTORC1 expression, suggesting synergistic activation of anabolic pathways. These findings highlight the potential benefits of combining resistance training with ursolic acid supplementation to mitigate diabetes‐induced muscle catabolism and preserve muscle mass. Therefore, this approach may be proposed as a potential therapeutic strategy to prevent muscle atrophy in diabetic patients. Further studies in diabetic human populations are warranted to validate these results.

### 5.1 Implications

These findings highlight the potential of combining resistance training with ursolic acid supplementation to counteract diabetes‐induced muscle catabolism in aged populations. The synergistic effect of the dual intervention on PI3K and mTORC1 signaling suggests a promising strategy for preserving muscle mass in diabetic patients. Future clinical trials are needed to confirm these benefits and optimize intervention protocols.

## Author Contributions

A.S., M.R., and M.F.: study design; A.S. and S.A.: protocol implementation and data collection; A.S. and S.A.: initial manuscript drafting; M.F., M.R., and R.K.T.: supervision and final revision.

## Funding

The authors have nothing to report.

## Ethics Statement

All experimental procedures were performed in accordance with the Guide for the Care and Use of Laboratory Animals and approved by the Animal Ethics Committee at Shahrekord University, Shahrekord, Iran (IR.SKU.REC.1399.001).

## Conflicts of Interest

The authors declare no conflicts of interest.

## Data Availability

Data supporting the findings of this study are available from the corresponding author upon reasonable request.
